# Feeding Behaviour, Swimming Activity and Boldness Explain Variation in Feed Intake and Growth of Sole *(Solea solea)* Reared in Captivity

**DOI:** 10.1371/journal.pone.0021393

**Published:** 2011-06-27

**Authors:** Julia Mas-Muñoz, Hans Komen, Oliver Schneider, Sander W. Visch, Johan W. Schrama

**Affiliations:** 1 Aquaculture and Fisheries Group, Wageningen University, Wageningen, The Netherlands; 2 Animal Breeding and Genetics Group, Wageningen University, Wageningen, The Netherlands; 3 Wageningen IMARES, Yerseke, The Netherlands; Roehampton University, United Kingdom

## Abstract

The major economic constraint for culturing sole (*Solea solea*) is its slow and variable growth. The objective was to study the relationship between feed intake/efficiency, growth, and (non-) feeding behaviour of sole. Sixteen juveniles with an average (SD) growth of 2.7 (1.9) g/kg^0.8^/d were selected on their growth during a 4-week period in which they were housed communally with 84 other fish. Selected fish were housed individually during a second 4-week period to measure individual feed intake, growth, and behaviour. Fish were hand-fed three times a day during the dark phase of the day until apparent satiation. During six different days, behaviour was recorded twice daily during 3 minutes by direct observations. Total swimming activity, frequency of burying and of escapes were recorded. At the beginning and end of the growth period, two sequential behavioural tests were performed: “Novel Environment” and “Light Avoidance”. Fish housed individually still exhibited pronounced variation in feed intake (CV = 23%), growth (CV = 25%) and behavior (CV = 100%). Differences in feed intake account for 79% of the observed individual differences in growth of sole. Fish with higher variation in feed intake between days and between meals within days had significantly a lower total feed intake (r = −0.65 and r = −0.77) and growth. Active fish showed significantly higher feed intake (r = 0.66) and growth (r = 0.58). Boldness during both challenge tests was related to fast growth: (1) fish which reacted with a lower latency time to swim in a novel environment had significantly higher feed intake (r = −0.55) and growth (r = −0.66); (2) fish escaping during the light avoidance test tended to show higher feed intake (P<0.1) and had higher growth (P<0.05). In conclusion, feeding consistency, swimming activity in the tank, and boldness during behavioral tests are related to feed intake and growth of sole in captivity.

## Introduction

Dover Sole (*Solea solea*) has a high potential for commercial aquaculture in Europe because of its consumer popularity and high market values [1], [2]. Currently, larvae of cultured sole are produced by natural reproduction of captured wild broodstock. Despite attempts for selective breeding and optimization of diets attractiveness, the species is still in an early stage of domestication. Possibly this explains the variable and low growth of sole in culture conditions, which remains one of the most important economic constraints for commercial sole in aquaculture [1], [2], [3], [4], [5].

Individual differences in growth are common in cultured animals, but fish generally show more pronounced variability than other livestock animals, with body weights ranging from 20–40% of the mean for most fish species [6]. Also in cultured sole, high growth variations have been reported, 30–50% for *Solea solea* [7] and 24–29% for *Solea senegalensis* [8].

Individual fish often show pronounced variation in both growth and behaviour within a group [9], [10], [11], [12], [13]. Most studies on individual differences in growth have focused on social interactions in groups of fish with social hierarchies as a major cause for growth heterogeneity [14], [15], [16], [17], [18], [19], [20]. Other studies have addressed the genetic component of growth rate distribution and the physiological mechanisms underlying growth variation of fish when held in isolation [10], [11], [16], [21]. Heritability values for body weight in sole and other fish species have been estimated with values ranging from 0.2 to 0.4 [6], [7]. In the absence of competition, where no social interactions exists, individual variation in growth would mainly indicate inherent inter- and intra-individual variability in feed intake, and feed efficiency (residual feed intake, RFI). Differences in residual feed intake are considered to be mainly due to differences in: basal metabolism and activity (maintenance costs), digestive efficiency (nutrient digestibility) and body composition (energy storage) [22]. In fish, feed utilization efficiency has been proven to have significant genetic variability [23]. Moreover, individual differences in feed consumption, can be caused by differences in feeding behaviour, such as day to day variation in feed intake or the feeding pattern within a day [24]. Individual differences in feeding strategies have been studied in Salmonid fish [25] and in Bluegill sunfish [26] which have been explained in terms of the changing trade-off between foraging and predator avoidance in nature [27], [28].

In nature, under predation risk, prey animals, such as young fish, face a conflict between two competing motivations: hunger and fear for predation. Studies on the foraging behaviour of prey species under predation risk show that individuals within a population show a continuum in their responses, from “bold” to “shy”, representing different strategies in terms of survival. “Bold” animals show active foraging behaviour regardless of predation risk, while “shy” animals try to limit predation risk at the expense of foraging [29], [30]. These coherent set of behavioural and physiological differences between individuals from the same population which are consistent over time and across situations are referred to as personality, coping styles, temperament or behavioural syndromes [31], [32]. “Bold or proactive” (active coping or fight-flight response) animals are often characterized by being more aggressive, explorative and more active in unfamiliar situations whereas “shy or reactive” (passive coping or conservation-withdrawal response) animals are considered to be more fearful or timid, and less active in the same situations [33]. These different “coping styles” result from genetic, environmental or ontogenetic factors and their interactions [34], [35], [36]. Previous studies have shown that innate behavioural and physiological traits represent different responses and adaptive strategies to environmental challenges [12]. Proactive individuals have a tendency to dominate and outcompete reactive ones in a stable environment with feed in excess. Nevertheless, the latter appear to respond better in an unpredictable or variable environment [31], [37].

In nature, selection pressures on behaviour may vary across time as it depends on environmental circumstances which coping type will be in advantage, thus variation in behavioural strategies is maintained [38]. Farmed fish reared in captivity have no accessibility to shelter, are reared at high densities, with predictable food delivery, and in the absence of predators thus, it is suggested that bold individuals with high competitive ability, more active and with risk-prone feeding behaviour display higher growth rates [37], [39]. Previous studies have reported positive associations between boldness and growth in captive or domesticated animals [27], [40], [41], [42].

Sole utilizes a detection minimization strategy to reduce predation risk in nature: they match the colour of sediment [43], spend long times buried in it [44], show low activity levels [45] and nocturnal feeding [46]. Therefore, it is hypothesized that individual variation in risk-prone feeding behaviour and activity (bold versus shy fish), may be related with the behavioural flexibility/capacity of fish to adapt to captive conditions and therefore explaining high individual differences in feed intake and thereby in growth of cultured sole.

This study aims to examine the inherent causes of individual variation in growth of sole *(Solea solea)*. The objective is to assess whether individual variation in feeding and non-feeding behaviour may explain differences in feed intake/efficiency and growth of sole reared in captivity.

## Materials and methods

### Ethics

All procedures involving animals were conducted in accordance with the Dutch law on experimental animals and were approved by the Ethical Committee for Animal Experiments (DEC) of Wageningen University.

### Experimental animals and housing

Juvenile sole (*Solea solea,* N = 100, not selected for sex) with an initial weight of 59.5±6.5 g were obtained from a local commercial farm (Solea BV, Ijmuiden, The Netherlands). Upon arrival fish were communally housed in one 400 L black tank of 2×1×0.4 m (L × W × H) connected to a RAS system. The RAS system consisted of two sludge settlers and one bio-filter containing lava rock filled with artificial sea water (25 ‰). Water temperature (17.8±0.1°C), pH (7.9–8.2), dissolved O_2_ (>7 mg/l), salinity (25± 0.1 ‰), NH_4_^+^ (<1 mg/ l) NO_2_^−^ (<1 mg/ l) and NO_3_^−^ (<50 mg/l) were monitored daily. Ammonia, nitrite, nitrate were maintained below this levels by exchanging sea water when necessary.

Fish were allowed to adapt to the experimental facilities for two weeks. After adaptation, they were individually weighted and PIT-tagged (Trovan ID100, DORSET GP, Aalten, Netherlands) while anesthetized with 2-phenoxyethanol (1 ml of solution/litre of system water).

The experimental period consisted of two growth periods, each of 28 days duration. During the first growth period, the 100 fish were group housed in the same 400 L tank. Based on the realized growth rate (GR, g/kg0.8/d) of this period, fish were categorized into eight different growth classes (Table 1). Two fish from each growth class were randomly selected. The 16 selected fish were individually housed in 30-L glass tanks (0.30×0.5×0.3 m) during a second growth period to measure individual feed intake and behaviour in the absence of social interactions. Tanks were connected to the same RAS systems as before and side walls were covered with black plastic to avoid any visual contact between fish. At the end of both growth periods fish were weighed and behavioural tests were performed.

**Table 1:** Classification of fish based on their growth in period 1^a^.

Growth Class	Growth Period 1(g/kg^0.8^/d)	Mean End BW Period 1 (g)^b^	Number of fish
1	<0.00	57.2	5
2	0.00–0.90	61.8	10
3	0.90–1.50	64.3	11
4	1.51–2.50	64.6	21
5	2.51–3.50	70.1	23
6	3.51–4.30	69.6	21
7	4.31–5.30	76.9	6
8	>5.30	84.7	3

a Two random fish per growth class were selected.

b BW = Body weight is averaged over the total number of fish categorized in each growth class.

A 12D:12L photoperiod was maintained using artificial fluorescent lights. As juvenile sole are nocturnal feeders [46] the light regime was reversed with lights on from 21:00 h till 9:00 h. During the dark period of the day red lights were used to provide sufficient light to feed and to perform video recordings.

### Feeding method

Fish were fed with a commercial feed diet, DAN-EX 1562 (DANA FEED A/S, Denmark, sinking pellet). Fish received 2 mm size pellets (61% protein, 20% fat and 24 kJ/g energy on dry matter basis) during period 1 and were switched to mm pellets (63% protein, 19% fat and 23 kJ/g energy on dry matter basis) during period 2 when fish had grown bigger.

During period 1 group housed fish were fed in access (between 0.5–1% body weight d^−1^) by an automated belt feeder, which distributed feed in two blocks of 3 hours. Feeding periods were from 9:00 h till 12:00 h and 13:00 h till 16:00 h. After each feeding all uneaten pellets were removed. To ensure feeding until apparent satiation daily rations were adjusted based on the feed intake of previous day.

The 16 individually housed fish in period 2 were hand fed three times a day at 8.00, 12.00 and 17.00 h until apparent satiation. For all fish the feeding period started with a feed ration of 15 pellets (0.27 g) and whenever pellets where eaten 5 extra pellets (0.09 g) were added. Through this procedure there would always be at least 5 pellets of feed in each tank during the feeding time. Feeding continued after pellet addition for a maximum of 20 minutes and five minutes later remaining pellets were siphoned and counted.

### Live behavioural observations

The behaviour of the16 fish housed individually was recorded by direct observations twice a day in between meals at Days 8, 10, 13, 15, 16 and 24 of period 2. In the morning observations were made between 10:30–11:00 h and in the afternoon between 15:00–16:00 h. Each fish was observed throughout a three minutes period during which total swimming activity (% of observation time), frequency of burying attempts on the bare bottom (#/3 min) and frequency of escapes (#/3 min) were recorded following the ethogram presented in Table 2. In total 12 observations per fish were made.

**Table 2:** Ethogram used for behavioural observations.

Behavioural element	Description	Live Observations	Novel Environment	Light Avoidance
Resting	Lying motionless on the bottom or against the side of the tank without performing any other described behaviour (state event)	x	x	x
Swimming	Displacement of the body using body or fin movement as propulsion (state event)	x	x	x
Small Movement	Fish moves slowly with no real displacement of the body, maximum distance covered is < half of fish length (state event)	x	x	x
Burying	Fish makes an attempt to bury by performing quick wave movements with its whole body (point event)	x	x	x
Escape	Fish moves its body straight up in the water column and is pushing its head out of the water surface (point event)	x	x	x
Activity	The total observation time minus the time spent resting	x	x	x
latency time to swim	Time elapsed from the time the fish went to rest for the first time until it performs any other active behaviour	-	x	-
Latency to go to dark	Time elapsed until the fish moves from Section A to Section B	-	-	x
Time in dark	Time the fish stays in the dark, section B	-	-	x

### Behavioural Tests

At the end of each growth period two sequential behavioural tests: “Novel Environment Test” and “Light avoidance Test”, were performed to each fish individually during the dark phase of the day. Red lights were used as illumination to allow video recording. Twenty-four hours prior to the behavioural tests fish were not fed to increase their potential activity.

The testing was performed in two successive rounds. During each round eight random fish were screened individually in eight 120 L (0.6×0.5×0.4 m) glass barren-bottom tanks at the same time for the conduction of the tests. The test-tanks were filled up to 20 cm with water from the RAS system and were refreshed completely at the end of each testing round to avoid chemical cues to interfere in the behavioural response. The test-tanks were visually isolated from each other by black acrylic sheets covering three sides of the tank. Each test-tank was divided into two equal sections (section A and B) by a plastic lid. Section A was open on the top and had a fluorescent light above, while section B was covered with a plastic lid on the top and was in complete darkness. Behavioural responses were recorded with two video cameras, one above and one on the side in section A of each tank.

During the “Novel Environment Test”, fish were restricted to section A. The test started with the introduction of the fish into the test-tank after which fish were monitored for 15 min. The reaction of each fish to this new environment was analysed following the ethogram in Table 2.

The second test, the “Light avoidance test”, started 45 minutes after the introduction of the fish into the test-tank. The test started with the opening of the connection to section B by lifting the plastic lid 12–15 cm and simultaneously increasing the light intensity in section A (approx. 600 Lux), whereas section B stayed dark (0 Lux). The behavioural response was recorded using the ethogram (Table 2) for a maximum of 15 minutes.

For each behavioural test the activity patterns were expressed as the percentage of total observation time. Burying and escapes bouts (frequency) were recorded during each test. Latency time to swim during the novel environment test and latency time to move towards section B during light avoidance test was measured as elapsed time in seconds from the time the test started. When no activity was performed at all during the 15 minutes of test, the fish was given as latency time a score of 15 minutes for statistical convenience. Total activity time was calculated as 100-Time resting (%).

Each test was performed twice with each fish, at the start and at the end of period 2. Due to technical problems (short-circuit) videos from 8 fish of the second testing day (end of period 2) were damaged and thus excluded from the analysis. Therefore, the data used for the behavioural analysis was the mean of all observations per fish. Video recordings from the behavioural tests were analysed using the “The Observer XT 9.0” software package (Noldus, Wageningen, The Netherlands).

### Data analysis

In the present study, fish were considered as experimental unit. Growth rate (GR) and feed intake (FI) were expressed per metabolic body weight as units of g BW (kg)^−0.8^ d^−1^. This was done to correct for the variation in fish size as it is known that larger fish have a greater absolute metabolic requirement of feed compared to smaller fish [47]. BW is the geometric mean of the weight calculated as:


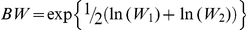
, where W_1_ is the initial weight (g) at the beginning of each growth period and W_2_ the end weight at the end of each growth period. Feed conversion ratio (FCR) was calculated by dividing total feed intake by weight gain during the period.

Feed efficiency was analysed using RFI (g/kg^0.8^/d). RFI was calculated as the difference between feed consumed by an animal and its consumption as predicted from a linear regression model involving the maintenance requirements and growth as independent variables FI = M +βGR +ε, where FI is the feed intake (g/kg^0.8^/d), M is the maintenance (g/kg^0.8^/d) and GR the growth (g/kg^0.8^/d) [48]. Animals with a low RFI (i.e. negative RFI) are assumed to be more feed efficient than animals with a high RFI (i.e. positive RFI).

Coefficient of variation (CV, %) was calculated as CV = 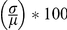, where σ is the standard deviation and μ the observation mean. The CV of feed intake between days (FIdays, %) was calculated using the standard deviation of FI between days and the average FI per day. The CV of feed intake between meals within days (FImeals, %) was calculated using the average standard deviation between meals in the day and the average FI per meal.

During the experiment one individually housed fish did not eat during period 2 (28 days starving) and was considered an outlier thus only data from 15 fish were included in the analysis.

Statistical analyses were performed using SAS system [49]. Data was analysed using linear regression models and performing Pearson's correlations between quantitative traits or if qualitative traits were defined significant effects were analysed using one-way analysis of variance (ANOVA) followed by the Turkey's HSD post-hoc test. The error terms of these analyses were tested for homogeneity of variances and normality, using the Levene's test and the Shapiro-Wilk test, respectively. Behavioural data was squared rooted (frequencies) or log transformed (latencies) when necessary. Results were considered statistically significant when p-values were below 0.05. Data is reported as mean ± SE.

## Results

### Growth during group housing conditions (Period 1)

The average growth of all the fish when group housed was 2.55±0.15 g/kg^0.8^/d (n = 100) and of the selected fish was 2.70±0.49 g/kg^0.8^/d (n = 15), displaying a wide range in growth (CV = 70%) during period 1. Body weight of selected sole at the end of period 1 was of 69.29±3.03 g (CV = 17%).

### Growth and feed intake/efficiency of individually housed sole (Period 2)

The average growth of the 15 individually housed fish was 5.2±0.3 g/kg^0.8^/d. Feed intake and FCR were of 4.3±0.3 and 0.84±0.03 g/kg^0.8^/d, respectively (mean ±SE, Table 3). No significant correlation was found between growth of individually housed sole and initial body weight (r = 0.17, P>0.1). The fish still exhibited pronounced variation in growth (CV = 25%) and feed intake (CV = 23%) during period 2.

**Table 3:** Pearson's correlations between growth, feed intake, feed efficiency (RFI), and behaviour of individually housed sole (n = 15).

			Pearson's correlations (r)		
Variable	Mean ± SE	CV (%)	Feed intake(g/kg^0.8^/d)	Growth(g/kg^0.8^/d)	RFI(g/kg^0.8^/d)
*Performance*					
Initial body weight (g)	69.8±3.1	17	0.24	0.17	0.20
Weight gain (g)	19.1±1.6	32	0.83***	0.89***	0.09
Growth P2 (g/kg^0.8^/d)	5.2±0.3	25	0.89***	1	0.00
Feed intake (g/kg^0.8^/d)	4.3±0.3	23	1	0.89	0.46+
FCR (g/g)	0.8±0.0	12	0.12	−0.33	0.90***
*Feeding behaviour*					
CV FI btw days (%)	33.2±4.8	55	−0.65**	−0.52*	−0.41
CV FI btw meals (%)	49.1±3.4	27	−0.77***	−0.64*	−0.45+
FI morning (% of daily FI)	38.1±1.2	12	−0.46+	−0.46+	−0.12
FI midday (% of daily FI)	27.1±1.0	14	−0.26	−0.12	−0.33
FI afternoon (% of daily FI)	34.8±1.1	13	0.71**	0.58*	0.42
*Activity home tank*					
Activity (%)	5.9±1.5	97	0.66**	0.58*	0.32
Escapes (#/3 min)	0.5±0.1	102	0.34	0.22	0.31
Bury (#/ 3min)	0.2±0.1	124	0.37	0.55*	−0.25
*Novel Environment test*					
Activity (%)	10.2±2.1	80	0.31	0.41	−0.11
Escapes (#/15 min)	6.0±2.7	172	0.33	0.24	0.26
Bury (#/15 min)	6.1±4.7	76	0.18	0.40	−0.39
latency time to swim (sec)	189.3±68.8	141	−0.55*	−0.66**	0.08
*Light avoidance test*					
Activity (%)	8.3±2.1	97	0.44	0.23	−0.34
Escapes (#/15 min)	0.9±0.4	170	0.46+	0.56*	−0.08
Bury (#/15 min)	4.2±1.5	137	−0.13	0.00	−0.26
Latency to move to dark (sec)	720.7±68.5	37	0.06	−0.03	0.19
Time dark (%)	16.4±68.5	125	0.23	0.22	0.09

Significant differences are indicated by

***p<0.001;

**p<0.01;

*p<0.05;

+p<0.1.

CV = Coefficient of variation, FI = Feed intake, FCR =  feed conversion ratio, RFI = Residual feed intake/feed efficiency.

**Figure 1:**
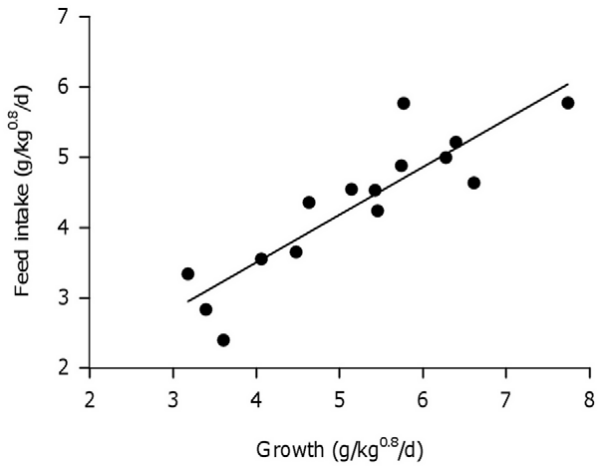
Relationship between feed intake (g/kg^0.8^/d) and growth (g/kg^0.8^/d) of 15 individually housed sole. (FI = 0.79 + 0.68*GR, R^2^ = 0.79, P<0.001).

The growth (GR, in g/kg^0.8^/d) of sole juveniles individually housed was strongly correlated to individual differences in feed intake (FI, g/kg^0.8^/d) and was described through the regression equation FI =  μ + β*GR + ε (μ = 0.79±0.52; β = 0.68±0.09; R^2^ = 0.79; P<0.001, Figure 1). According to the estimated linear regression on average 79% of the individual variation in feed intake was explained by variation in growth. The remaining 21% of variation in feed intake is the residual feed intake (RFI, g/kg^0.8^/d) which represents individual differences in feed efficiency and measuring errors. The average maintenance ration (μ), feed intake at which growth is zero obtained from the regression line, was 0.79±0.52 g/kg^0.8^/d.

### Relationship between feeding behaviour and feed intake/efficiency and growth

The feed intake of individually housed sole showed high variation between days and between meals within days with CV = 55% and 27%, respectively (Table 3). Differences in day to day variation in feed intake ranged from 14–85% and variation in feed intake between meals within a day varied from 38–75%.

**Figure 2:**
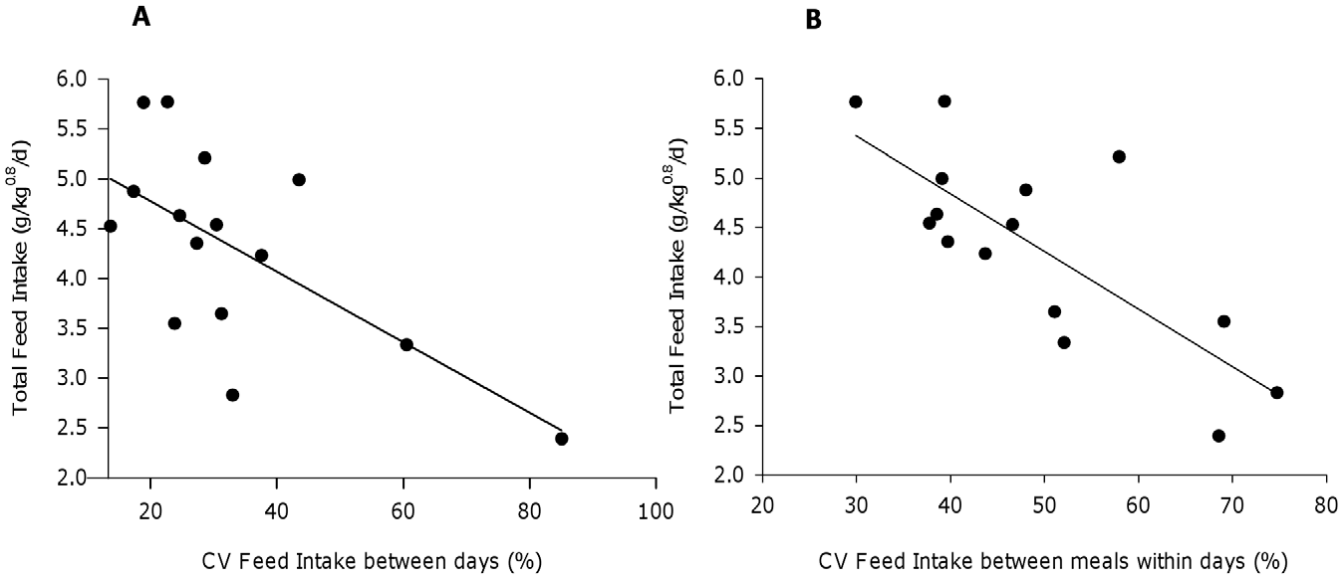
Relationship between total feed intake (g/kg^0.8^/d) and the CV of feed intake between days (A) and between meals within days (B). Regression equations are A) y = 5.49−0.04× (R^2^ = 0.43, P<0.01) and B) y = 7.17−0.06× (R^2^ = 0.60, P<0.001).

The CV of feed intake between days (FIdays, %) and between meals within days (FImeals, %) was negatively correlated with feed intake (g/kg^0.8^/d) of sole (FI = 5.49–0.04*FIdays, R^2^ = 0.43; P<0.01 and FI = 7.17–0.06*FImeals, R^2^ = 0.60; P<0.001, Figure 2). Correspondingly a significant negative correlation was found between the CV of feed intake and growth (g/kg^0.8^/d) (Pearson's correlations with FIdays and FImeals of r = −0.52 and r = −0.64, P<0.05, Table 3). However, no significant correlations were found with feed efficiency (RFI, g/kg^0.8^/d) (P>0.1, Table 3).

The feeding pattern within day showed that during the three meals given at 9:00, 12:00 and 17:00 h, sole consumed on average 38.1±1.2, 27.1±1.0 and 34.8±1.1% of their total FI respectively (means ± SE, Table 3). Fish which showed a higher percentage of feeding at 17.00 h had higher feed intake (r = 0.71, P<0.01) and growth rate (r = 0.58, P<0.05) during period 2. Whereas fish which showed high feeding levels during the first meal of the day (9:00 h) tended to have a lower total feed intake and growth (r = −0.46, P<0.1, Table 3). The percentage of FI during the midday meal (12:00 h) was significantly lower than the other two meals (P<0.05) and showed no significant relationship with feed intake or growth.

### Relationship between swimming activity and feed intake/efficiency and growth

**Figure 3:**
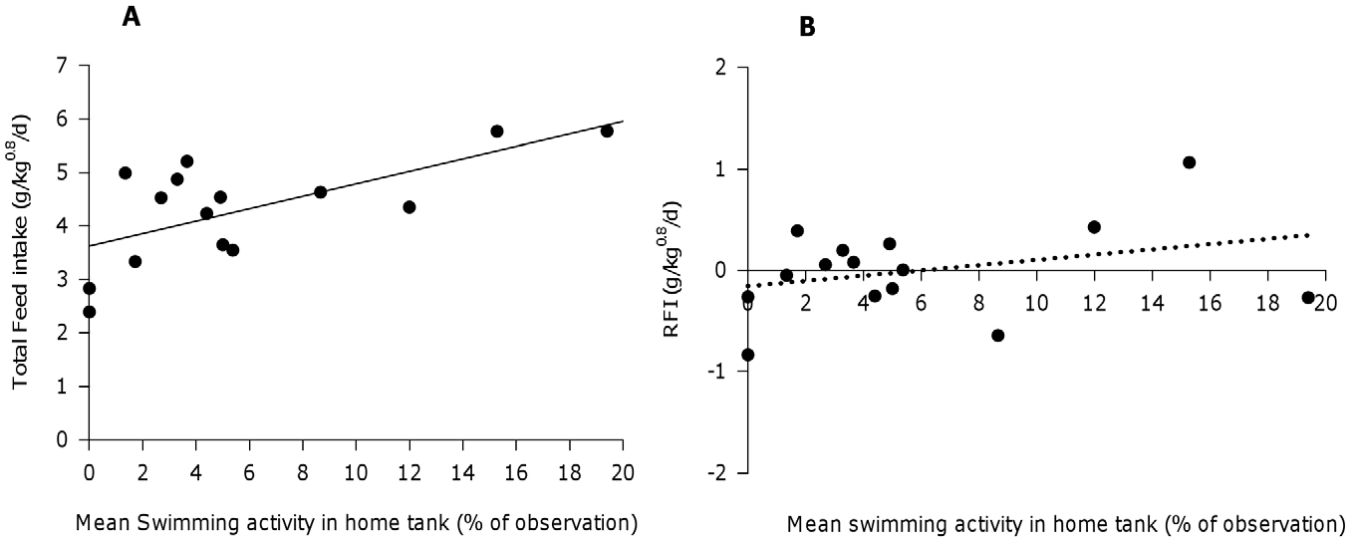
Relationship between swimming activity (%) in the home tank and A) total feed intake (g/kg^0.8^/d) and B) residual feed intake (g/kg^0.8^/d) of 15 individually housed sole. Regression equations are A) FI = 3.63+0.12*SWIM (R^2^ = 0.44, P<0.05) for feed intake and B) RFI = −0.15+ 0.03*SWIM (R^2^ = 0.10, P>0.1) for residual feed intake.

Feed intake was positively correlated with the average swimming time (SWIM, in %) during live observations in the tank (in between feeding periods) (FI = 3.63+0.12*SWIM, R^2^ = 0.44, P<0.05). Moreover, feed efficiency was not affected by differences in swimming activity (RFI = −0.15+0.03*SWIM, R^2^ = 0.1, P>0.1, Figure 3). Correspondingly, a positive correlation was found with growth (r = 0.58, P<0.05, Table 3). Active swimmers were also feeding more consistently with a significantly lower CV of feed intake between meals within days (r = −0.61, P<0.05) and a trend for lower CV of feed intake between days (r = −0.47, P<0.1, Table 3).

Sole that were escaping during live observations (n = 10 fish) had higher feed intake than fish which did not (n = 5 fish) (FI: 4.7±0.3 versus 3.6±0.4 g/kg^0.8^/d, P<0.05, Table 4). However, fish escaping during observations also tended to be less feed efficient compared to those that were not seen displaying this behaviour (RFI: 0.1±0.1 vs. −0.3±0.2 g/kg^0.8^/d, P<0.1, Table 4) but no significant differences were found regarding growth (P>0.1, Table 4).

**Table 4:** Comparison of growth, feed intake and feed efficiency (RFI) between fish displaying escape behaviour (present vs. absent)^b^.

	Home tank observations			Novel environment test			Light avoidance test		
Variable	Escape(n = 10)	No Escape(n = 5)	P	Escape(n = 10)	No Escape(n = 5)	P	Escape(n = 5)	No Escape(n = 10)	P
Growth P2(g/kg^0.8^/d)	5.5±0.4	4.6±0.6	ns	5.4±0.4	4.7±0.6	ns	6.1±0.5	4.7±0.4	*
Feed intake (g/kg^0.8^/d)	4.7±0.3	3.6±0.4	*	4.5±0.3	4.0±0.5	ns	4.9±0.4	4.0±0.3	ns
RFI^a^(g/kg^0.8^/d)	0.1±0.1	−0.3±0.2	+	−0.01±0.15	0.02±0.21	ns	−0.06±0.21	0.03±0.15	ns

Values are means ±SE. Significant differences are indicated by;

*p<0.05;

+p<0.1; ns = not significant.

aRFI =  Residual feed intake/feed efficiency.

bClassification of the fish differs between observations in the home tank, the Novel environment test and the Light avoidance test.

The frequency of burying in the barren tank during live observations was positively correlated with growth but not with feed intake (r = 0.55, P<0.05 and r = 0.37, P>0.1, Table 3).

### Boldness during behavioural tests and its relationship with feed intake/efficiency and growth

Results from the challenge behavioural tests show high individual variation in behavioural responses, CV of behavioural traits ranging from 36–170% (Table 3). Behavioural tests were relatively consistent in time with Pearson's correlation of individual behavioural responses between both testing periods ranging from 0.4–0.7. Individual's responses to novelty and to light showed to be related with their feed intake and growth in captivity.

#### 1. Novel environment test

Sole responded to a new environment with a swimming activity of 10.2±2.1% of observation, with on average of 6.0±2.7 escapes and 6.1±4.7of burying attempts (Table 3). The average latency time to swim and to start exploring the new environment (as a measure of boldness) was 189±69 sec, and was negatively correlated with total feed intake (r = −0.55, P<0.05) and growth (r = −0.66, P<0.01, Table 3). Sole which responded with escaping (n = 10 fish) did not show a significance difference in growth compared to sole which did not escape (n = 5 fish) (P>0.1, Table 4).

#### 2. Light avoidance test

Sole subjected to the “Light avoidance test” showed in the illuminated area an average activity of 8.3±2.1% of observation, and displayed on average 0.9±0.4 escapes and 4.2 ±1.5 burying attempts. The average latency to go to the dark section of the tank (section B) was 720.7±68.5 seconds and the total time in the dark was of 16.4±5.3% (Table 3). Sole showed two opposite coping styles when exposed to a high light intensity: 1) Proactive fish which escaped and, 2) Reactive fish which remained in the bottom. The frequency of escaping during the light test tended to be positively correlated with feed intake (P<0.1, Table 3) and significantly with growth (P<0.05, Table 3). However, no significant relationship was found with feed efficiency (P>0.1, Table 3). Sole which responded with escaping (n = 5 fish) had a higher growth compared to sole which did not escape (n = 10 fish) (6.1±0.5 vs. 4.7±0.4 g/kg^0.8^/d, P<0.05, Table 4).

## Discussion

The present study showed that sole *(Solea solea)* housed individually, in the absence of social interactions, still exhibits high individual differences in feed intake, growth and behaviour (on average CV of 23, 25 and 100% respectively), which has also been observed in other species when held in isolation [9], [10], [11], [50]. Growth variation of sole housed individually was lower than when communally held in a group of 100 fish (CV was 25% in period 2 compared to 70% in period 1, P<0.05). This results are in line with studies in other fish species were the reported variation in feed consumption within grouped fish showed a marked increase compared to variation in feed intake when fish were held in isolation, on average 60–100% versus 25–40% [16], [24], [51], [52]. There are three main causes of phenotypic variation among individuals in a population: 1) genetic; 2) environmental; 3) interaction between genetic and environmental factors. In this study, individual differences were measured in the absence of social interactions and with equal and predominantly constant environmental conditions, which suggests that differences in feed intake, growth and behaviour have a genetic basis.

This study showed that under ad libitum conditions and individual housing, differences in feed intake account for 79% of the observed individual differences in the growth of sole. These results are in agreement with other studies in fish showing that the variation in the growth of fish is mainly due to variation in feed intake [16], [19], [24], [53]. Data on individual feed intake of sole on dry feed is limited in literature and generally difficult to compare as feed intake depends on the respective diet nutrients, feeding protocol, size of the fish, temperature and origin of the fish. However, our results (4.3 g/kg^0.8^/d or 0.87%/d) are comparable to feed intake of grouped housed Solea solea of similar weight class from studies of S. Ende et al., 2009 (Personal communication) and [54]. Mean growth rate in this study (5.2 g/kg^0.8^/d or 0.86%/d) is within the higher level of displayed growth of grouped housed Solea solea with values in literature ranging from 0.86–0.3%/d [54], [55], [56].

Feeding behaviour was expressed as individual differences in feeding consistency over time and the daily feeding pattern: the coefficient of variation of intra-individual feed intake between days and between meals in the day was measured. A low CV indicated that the meal size or feed intake of an individual fish was similar from day to day and/or between the daily meals whilst a high CV indicated a more varied feed intake. Variation in feed intake between days is caused by a combination of endogenous and exogenous factors which can influence appetite and it appears to be a common feature of feeding in fish [24], [57]. The observed individual variations in CV of feed intake between days (14–85%) in sole were quite high compared to studies in other species held in isolation such as carp, with ranging values of 16–22% [24] or with minnows with values ranging from 21–27% [58]. Moreover, the present results show that fish which feed more consistently over time (within day and over days), show higher feed intake and growth but also tend to be less feed efficient. The influence that the regularity of feeding has on growth and feed efficiency is yet not well understood. However, it has been reported that the rate of protein synthesis is correlated with growth, which accounts for large proportion of total energy costs in fish and thus contributing to individual variations in growth efficiency [22], [59]. These findings are in accordance with a study in grass carp where fish with larger variability in feed intake had lower growth rates and hence lower rates of protein synthesis [24]. Recent studies also reported that differences in feed efficiency (residual feed intake) were related to the feeding motivation in African catfish [11] and to feeding activity in Nile Tilapia [53]. Additionally, we found that the daily feeding pattern also explained variation in feed consumption and growth of sole. Fish which showed a higher percentage of feeding during the last meal of the day (afternoon meal at the end of dark period, 17.00 h) had higher feed intake and growth. In line with our results, studies on other flatfish showed that individual variations in the feeding behaviour of halibut were stable across time and situations and were related to feed intake and growth [12]. The feeding rhythm of sole can be described by two major meals: one in the morning and one in the afternoon (at the beginning and the end of the dark period), where feed intake was significantly higher than during the midday meal. These results agree with other studies in which juvenile sole were found to have two main activity/feeding peaks during the night, one at sunset and another shortly before dawn [46], [60]. Other species, such as Atlantic salmon, also show feed intake peaks during the early morning and late afternoon [61].

Results suggests that for sole endogenous factors already explain high individual differences in food consumption, which indicate consistent differences in feeding strategies between individuals. Individual differences in feeding behaviour could be related to differences in the behavioural flexibility (or adaptive capacity) between fish to feed and grow in captivity, where coping styles might play an important role, as bold or active fish were also found to feed more consistently.

Active sole had significantly higher feed intake and growth, which agrees with results on Chinese sturgeon [10]. Activity time was not correlated with feed efficiency (RFI), thus individual differences in maintenance costs due to different activity levels in sole seem to have a relative small effect on RFI. In accordance, other studies highlighted that flatfish probably spend relatively less energy in swimming and allocate more food energy on growth than (pelagic) round fish [62]. The high feed intake of active fish might be due to the fact that individuals that spend more time swimming have higher appetite and increase their feed intake which may overcompensate differences in maintenance costs. Another explanation for this can be that more active individuals are often seen as better competitors, expropriating resources from less active individuals [30]. Fish displaying escaping behaviour at the water surface also showed a higher feed intake, however tended to be less feed efficient and no differences in growth were found. This type of behaviour is considered to be indicative for abnormal or stereotypic behaviour in flatfish (reflecting a stressed state of the fish). Contrary to this, findings in Atlantic halibut showed that surface swimming was an indicator for low growth rate [63]. However, surface swimming in this case was a combination of escapes and swimming close to water surface as it was measured with a pit antenna, thus the behaviour measured is a different behavioural trait. Furthermore, halibut were group housed, so this behaviour could have also been triggered by social interactions. Both behaviour and housing conditions were different, thus the comparison between results from both studies is difficult.

Moreover, boldness of sole measured as the reaction to an unknown/novel environment and to a sudden increase in light intensity proved to be related to feed intake and growth but not with feed efficiency (RFI). Sole which resume activity earlier in a novel environment and those that reacted escaping when confronted with a light stimulus had higher feed intake and growth. These results suggest that individual differences in behaviour when confronted to environmental challenges explain individual variations in feeding behaviour and growth, where proactive sole seem to be more successful in their feeding behaviour and thus display higher growth under captive conditions. Accordingly, animal personality traits, such as boldness, activity and aggressiveness have been reported in many species and have been found to be also positively correlated with feed intake or growth in captivity: Wilson et al.(1993) developed the shy-bold continuum for juvenile pumpkinseed sunfish with positive correlations between predator inspection, speed acclimation to the laboratory, foraging behaviour and parasitic infection [33]. Boldness towards predators was also positively correlated with growth and dispersal in killifish [29] and activity, foraging and growth in larval salamanders [64]. Salmonid fish also show individual variation in behaviours such as space use [65], boldness [42], and aggressiveness [66] where behavioural characteristics proved to be related with growth differences [41], [42] . Studies on Paradise fish, found that behavioural responses to a Novel environment were highly inherited [35], [36]. Thus, as coping styles seem to have a genetic base [32] these results suggest that selecting for growth in fish under such conditions will promote risk-prone feeding behaviour and high activity in tanks.

### Conclusion

The wide inherent individual variations in behaviour, feed intake and growth of sole suggest scope for improvement in sole aquaculture. Individual differences in feeding consistency, swimming activity and behavioural reactions under challenging situations (novel environment; increased light intensity) explain variations in feed intake and growth. Both feeding consistency and escaping behaviour also tended to explain differences in feed efficiency (RFI). These results suggest the existence of coping styles in sole which can influence their adaptive capacity to farming conditions: Proactive fish seem to have a more successful feeding strategy in captivity, displaying higher feed intake and growth . Therefore, behavioural traits may be of interest to have into account for selection in breeding programs. Additionally, high feed intake was related with the presence of more escaping behaviour which has been considered to be stereotypical behaviour in flatfish (reflecting a stressed state of the fish) which might be of importance when considering welfare and performance of fish in captivity.
